# Polycyclic aromatic hydrocarbons bound to outdoor and indoor airborne particles (PM2.5) and their mutagenicity and carcinogenicity in Silesian kindergartens, Poland

**DOI:** 10.1007/s11869-016-0457-5

**Published:** 2016-12-24

**Authors:** Ewa Błaszczyk, Wioletta Rogula-Kozłowska, Krzysztof Klejnowski, Izabela Fulara, Danuta Mielżyńska-Švach

**Affiliations:** 1 Environmental Toxicology Group, Institute for Ecology of Industrial Areas, 6, Kossutha St., 40-844 Katowice, Poland; 2 Department of Air Protection, Institute of Environmental Engineering, Polish Academy of Sciences, 34, Skłodowskiej-Curie St., 41-819 Zabrze, Poland; 3 Central Laboratory, Institute for Ecology of Industrial Areas, 6, Kossutha St., 40-844 Katowice, Poland; 4 Nursing Institute, Witold Pilecki State School of Higher Education, 8, Kolbego St., 32-600 Oświęcim, Poland

**Keywords:** Outdoor, Indoor, PM2.5, PAHs, Diagnostic ratios, MEQ, TEQ

## Abstract

Assessment of exposure to polycyclic aromatic hydrocarbons (PAHs) is important due to the widespread presence of PAHs in the environment and their toxicological relevance, especially to susceptible populations such as children and their health. The aim of this study is to compare indoor and outdoor concentrations of particulate matter with a diameter of 2.5 μm or less (PM2.5) and 15 individual PAHs, as well as contribution of the analyzed PAHs to mutagenic and carcinogenic activity. Samples were collected during spring season in two sites in southern Poland (Silesia) representing urban and rural areas. Indoor samples of PM2.5 were sampled in kindergartens. At the same time, in the vicinity of the kindergarten buildings, the collection of the outdoor PM2.5 samples was carried out. Mutagenic (MEQ) and carcinogenic (TEQ) equivalents related to BaP and the percentage share expressed as mutagenic (MP) and carcinogenic (CP) potential of each individual compound to the total mutagenic/carcinogenic potential of the PAH mixture were calculated. The obtained results show that high concentrations of PM2.5 (above 25 μg/m^3^) and 15 PM2.5-bound PAHs in outdoor and indoor air were similar in the two studied areas. In overall PAHs mutagenic and carcinogenic potential, the percentage share of benzo(a)pyrene (BaP) was dominant and varied from 49.0–54.5% to 62.5–70.0%, respectively. The carried out study indicates the necessity of reducing PAH emission from solid fuel combustion, which is reflected in PM2.5-bound PAHs concentrations and their diagnostic ratios. In the recent years, health effects on children resulting from their activity pattern and air quality in the public places have been a serious problem.

## Introduction

Air pollution is a major environmental health problem affecting everyone in developed and developing countries alike. It is estimated that worldwide in 2012, air pollution was the cause of 3.7 million premature deaths per year. This mortality is due to exposure to atmospheric particulates of 10 μm or less in diameter (PM10), which causes cardiovascular and respiratory diseases and cancers (WHO 2014).

Total human exposure to air pollutants is determined by the concentrations found in different microenvironments and time spent in each of them, commonly called the time activity pattern*.* People spend more than one half of their time indoors, with variations attributable to age, gender, and place. Although the fraction of time spent indoors is lower in rural than that in urban areas, individual exposures are often huge due to high concentrations of pollutants in the indoor air. Therefore, indoor exposure, including that in occupational settings, dominates in the total exposure to different pollutants (Perez-Padilla et al. 2010).

Pollution of the air with various substances depends on the emission source. Outdoor pollutants in the atmospheric air mostly came from anthropogenic sources, such as combustion of different kinds of fuel predominantly present in industrial processes and road traffic. Additionally, such processes as chemical reactions and transformations, dispersion, as well as long range transport in the atmosphere and local meteorological conditions also affect the air quality (Vallero 2014). Indoor pollution may result from combustion processes for cooking and heating, inflow of outdoor air pollutants, human activities (such as smoking, presence of biological agents, and use of chemical substances), consumer products and emissions from construction materials or furniture, as well as improper maintenance of ventilation or air conditioning systems (Perez-Padilla et al. 2010).

Especially dangerous for human health with well-established mutagenic and carcinogenic properties are polycyclic aromatic hydrocarbons (PAHs) and their nitro-, amino-, and other derivatives. The widespread prevalence of PAHs in the environment (air, water, soil, and sediments) as well as in diet, tobacco smoke, and some skin pharmaceutical products is connected with their formation during the incomplete combustion or pyrolysis of organic materials, such as coal, oil, or wood. PAHs are relatively poorly soluble in water and highly lipophilic; therefore, their bioavailability after ingestion and inhalation is significant. All these factors determine the persistence and capacity of PAHs to be bioaccumulated in the lipid tissue of plants and animals belonging to the food chain (IARC 2010).

PAHs in the environment represent a significant risk to human health, due to their intake into the body through the respiratory system, by ingestion of food or through the skin. Exposure to PAHs may cause short- and long-term health effects, in particular, connected with respiratory and cardiovascular symptoms or diseases (Perez-Padilla et al. 2010; WHO 2014). Unmetabolized PAHs can have toxic effects, but after bioactivation to electrophilic metabolites, they demonstrate mutagenic or carcinogenic effects. A major concern is the ability of the reactive metabolites, such as epoxides and dihydrodiols, of some PAHs to bind to cellular proteins and DNA and adduct formation (Balbo et al. 2014). Biochemical disruptions and cell damage occurrence lead to mutations, developmental malformations, tumors, and cancers. The recent data indicate that benzo (a) pyrene (BaP) and mixtures of PAHs, such as tobacco smoke or gases from household combustion of coal, and exhausts from diesel engine are classified to group 1 as carcinogenic to humans (IARC 2016).

In the air, PAHs like other volatile and semi-volatile pollutants adsorb on suspended particles (particulate matter, PM). Atmospheric PM is a complex mixture of extremely small particles and liquid droplets made up of a carbonaceous fraction and inorganic constituents (Rogula-Kozłowska 2016). In the recent years, road traffic has become one of the major sources of PM in urbanized regions all over the world. The influence of traffic emissions on PM consists mainly in enriching PM in carbon compounds which is clearly visible in the case of fine PM (Rogula-Kozłowska 2016).

What is very significant in human exposure to PM is the size of particles, due to their harmful effects to the organisms. Fractions of particles with a diameter of 2.5 μm or less (PM2.5, fine PM) are able to penetrate deeply into the lungs and deposit in the alveoli. As studies showed, daily exposure to particulate matter is associated with increased incidences of hospital admissions, chronic asthma, and premature death, as well as respiratory problems in children (Perez-Padilla et al. 2010).

Potential risk and characterization of a complex mixture of PAHs can be identified using toxic equivalency factors (TEF) based on BaP. Biological activity of individual PAHs relative to BaP has been previously considered by Nisbet and LaGoy (1992) and Durant et al. (1996). Similarly, the replacement of TEF with mutagenic equivalency factor (MEF), also related to BaP, was calculated (Rogula-Kozłowska et al. 2013a). Currently, an increasing number of papers concerning this approach can be observed, both for carcinogenic and more often mutagenic properties of PAHs (Delgado-Saborit et al. 2011; Masiol et al. 2012; Krugly et al. 2014; Hassanvand et al. 2015).

In the present study, fine particulate matter PM2.5 was collected in two kindergartens and the nearby air monitoring stations in outdoor and indoor environments in Silesia (Poland). Silesia seems to be one of the most polluted regions in Europe (Rogula-Kozłowska et al. 2013a, 2013b, 2014). There is an interesting contrast between the origin of ambient particulate matter in Western Europe and in Poland, especially in highly urbanized and industrialized Upper Silesia, where municipal and industrial emissions and emissions from energy production overshadow emissions from traffic (Rogula-Kozłowska et al. 2013a, 2013b). On the other hand, the structure of air pollution in Poland is representative of Central and Eastern European countries, where the energy production relies on combustion of fossil fuels (mainly coal). The study was focused on the determination of concentrations of 15 selected PM2.5-bound PAHs and assessment of their properties recognized as mutagenicity (MEQ) and carcinogenicity (TEQ), also expressed as mutagenic (MP) and carcinogenic (CP) potential. The origin of PM2.5-bound PAHs was identified based on the diagnostic ratios.

## Materials and methods

### PM2.5 sampling - sites and methods

**Fig. 1:**
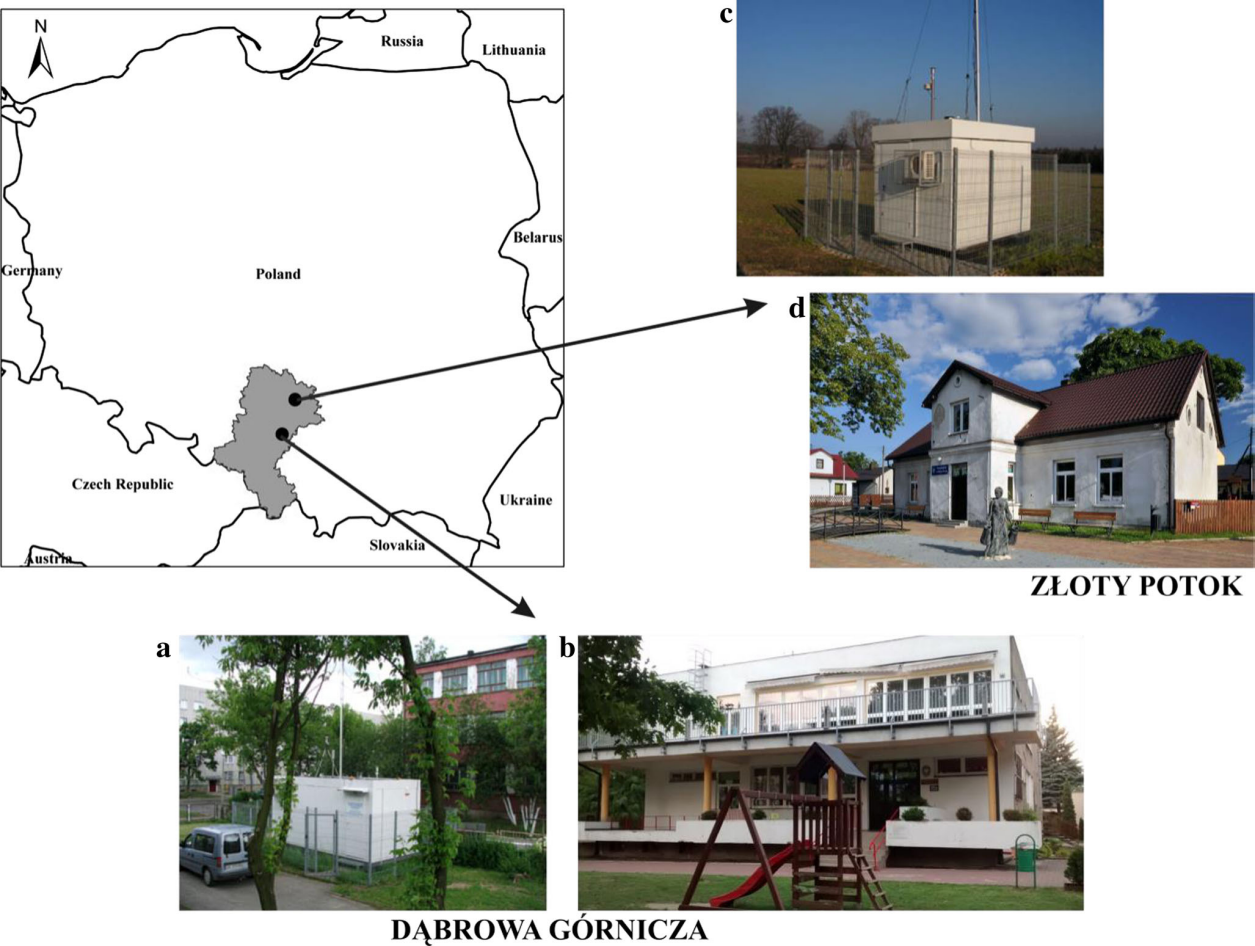
Localization of sampling sites in Silesian voivodeship, Poland. Photographs present outdoor air monitoring station (**a**), kindergarten in Dąbrowa Górnicza (**b**), outdoor air monitoring station (**c**), and kindergarten in Złoty Potok (**d**)

Outdoor and indoor samples of PM2.5 were collected at two sites in southern Poland (Fig. 1). The first site represents an urban-industrial area in Dąbrowa Górnicza and the second one - a rural region in Złoty Potok. The sampling point in Dąbrowa Górnicza (geographic coordinates: latitude 50.329111° North, longitude 19.231222° East, elevation 281 m above sea level) may be considered as an urban background site according to Directive (2008). Dąbrowa Górnicza is located in the Upper Silesian Industrial Region, where like in other cities in this region - and opposite to other parts of Poland - there are some industrial plants, including steel mills, coke plants, glass and plastic manufacturing companies, etc. The sampling site in Złoty Potok (geographic coordinates: latitude 50.710889° North, longitude 19.458797° East, elevation 291 m above sea level) localized in the commune of Janów represented the regional background site according to Directive (2008). It was surrounded by meadows, arable lands, and forests. Location of the described sampling points is presented in Fig. 1.

At both sites, PM2.5 samples were collected in parallel outdoor and indoor. Indoor PM2.5 was sampled in the kindergartens. The kindergarten in Dąbrowa Górnicza was located in a two-storey, detached building with six classrooms, a locker room, and a kitchen. The kindergarten in Złoty Potok was smaller and situated in a single-storey, detached building with one didactic room and one room where children used to play and eat meals. A detailed characteristic of both kindergartens is presented in Table 1. Outdoor PM2.5 samples were collected in the vicinity of the kindergarten buildings. The sampling points were regional air quality monitoring stations (Fig. 1). Location of these stations meets the requirements for urban background and regional background sites defined in Directive (2008).

**Table 1:** Detailed characteristics of kindergarten buildings

Parameter	Kindergarten	
	Dąbrowa Górnicza	Złoty Potok
Number of children (group)	125 children (six groups)	25 children (one group)
Heating system	Central heating from the municipal network	Electric heating
Window frame material	Plastic and wood	Wood
Kitchen stove type	Gas stove	Gas and coal stove
Floor type	PCV flooring	Carpets and laminate wood floor
Ventilation system	Gravitational	Gravitational

Outdoor and indoor samples of PM2.5 were collected during the spring season (17 March–09 April 2010 in Złoty Potok and 10 April–3 May 2010 in Dąbrowa Górnicza). In the southern cities of Poland during spring season, beside typical sources of PM (e.g., transport, industry, power plants, etc.), PM sources associated with individual heating of homes, buildings, etc. occur. For this reason, spring season can be recognized as a heating period. On the other hand, in southern Poland during spring season, unlike during winter season, no so called smog episodes are observed, which due to the high level of air pollution and short duration, exclude the winter season from comparative experiments, such as those described in the work of Rogula-Kozłowska et al. (2013b, 2014).

The atmospheric conditions during measurements were characteristic for the spring period in southern Poland. The average air temperature in this time range from 2 °C (in March) to 8 °C (in May). The atmospheric pressure was more or less balanced and oscillated within 975–985 hPa. The average wind speed was in the range of 0.2–3 m/s, and the relative humidity fluctuated between 70 and 99%. Detailed information about meteorological conditions in both locations and the variability of these conditions over several years are available on the website of the regional air quality monitoring for the Silesian region (http://powietrze.katowice.wios.gov.pl/archiwalne-dane-pomiarowe).

In this study, in total, 40 24-h samples of PM2.5 were collected (20 24-h samples in Dąbrowa Górnicza and Złoty Potok, respectively). In each location, 10 PM2.5 samples were collected in kindergartens (indoor sample) and 10 PM2.5 samples of atmospheric air (outdoor samples) - in regional air quality monitoring stations. The sampling was performed when kindergartens were open, i.e., at the presence of children.

Sequential Dichotomous Partisol-Plus (Model 2025) was used for outdoor 24-h PM2.5 sample collection (air volume 1 m^3^/h). The 24-h PM2.5 samples of indoor particles were taken with a PNS-15 sampler (Atmoservice LVS, air volume 2.3 m^3^/h). Before the air sample collection, concentrations measured simultaneously using the two samplers were compared, and it was observed that the obtained results were consistent (on average, for the 14 24-h measurements, the difference in the concentrations measured by these samplers was 0.8 g/m^3^). All samples of PM2.5 were collected on quartz fiber filters (QMA, 47 mm in diameters). Before and after the exposure, the filters were conditioned in a weighing room (48 h; relative humidity 45 ± 5%; air temperature 20 ± 2 °C). Then, they were weighed on the Mettler Toledo microbalance (resolution 2 μg). The methodology for conditioning, weighing, storage, and transport of samples as well as blank sample preparation complied with the QA/QC procedures defined in the reference method for gravimetric measurements (EN 12341:2014). The weighing accuracy, determined as three standard deviations from the mean obtained from 10 weighings of a blank filter (conditioning performed every 48 h), was 20.5 g. After weighing, the exposed filters were put into Petri dishes which were wrapped in light-proof aluminum foil and stored in a freezer at −18 °C till the PAH analysis.

### PAHs analysis

Concentrations of PM2.5-bound naphthalene (NP), acenaphthene (ACE), fluorene (FL), phenanthrene (PHE), anthracene (ANT), fluoranthene (FLA), pyrene (PYR), benz(a)anthracene (BaA), chrysene (CHR), benzo(b)fluoranthene (BbF), benzo(k)fluoranthene (BkF), benzo(a)pyrene (BaP), dibenz(a,h)anthracene (DahA), benzo(g,h,i)perylene (BghiP), and indeno(1,2,3-c,d)pyrene (IcdP) were identified according to standard methodology (EN 15549:2008). Each sample of PM2.5 deposited on the filter was extracted in an accelerated solvent extractor (ASE 350, Dionex) with dichloromethane (DCM) as an extraction solvent. The extraction procedure parameters were as follows: temperature 100 °C, pressure 10 Mpa, and time 30 min. The extracts were placed in a water bath at 27 °C and concentrated to approximately 1 ml with nitrogen gas in concentration workstations (TurboVap, Caliper LifeSciences). The concentrated extract was purified on a Florisil column filled with 60–100 mesh. PAH fraction was eluted with DCM, using 10 ml DCM per 1 g of Florisil. The eluates were concentrated again in TurboVap to a volume of 1 ml. Analysis of the extract was carried out using high-performance liquid chromatography with a fluorescence detector (HPLC-FLD 1200, Agilent Technologies). PAH resolution was performed using RP C18 column (PAH LiChrospher 5 μm × 250 × 3 mm, Merck) and gradient elution: water-methanol.

PAHs were determined qualitatively and quantitatively using a fluorescence detector (FLD), with programmable time changes in wavelength of the excitation and emission for NP, ACE, and FL – 220/330 nm; PHE, ANT, FLA, PYR, BaA, and CHR – 260/420 nm; BbF, BkF, BaP, DahA, and BghiP – 290/450 nm; and IcdP – 248/500 nm. Calibration of the chro-matography system was performed using five standard solu-tions in the range of 0.01–1 μg/ml. Calibration curves (peak area against the compound concentration) were linear, with a correlation coefficient of more than 0.998; precision in the whole concentration range did not exceed 20%. Quality con-trol was conducted by the study of blank samples and deter-mination of the recovery coefficients for marked PAHs (aver-age recovery coefficient was 0.955–1.081). As a reference material, the sample of urban dust was used (SRM 1649b). For the calibration of HPLC-FLD, the certified analytical stan-dard PAH mixture 100 μg/ml in dichloromethane (Ultra Scientific PM-611-1) was applied. Detection limits for the developed PAH determination method in PM air samples var-ied from 0.012 to 0.017 ng/m^3^.

### Mutagenicity and carcinogenicity of PAHs

Mutagenic (MEQ) and carcinogenic (TEQ) equivalents in ng/m^3^ were calculated by multiplying the concentrations of each PAH compound with its mutagenic equivalency factor (MEF) for mutagenic potential relative to BaP and carcinogenic equivalency factor (TEF) for cancer potential relative to BaP, respectively. MEQ and TEQ levels for the sum of PAHs were calculated using appropriate values of MEF and TEF (Table 2) according to the following equations (Eqs. 1–2):

1 $$ MEQ=\sum_{i=1}^N{(PAH)}_i*{MEF}_i $$

2 $$ TEQ=\sum_{i=1}^N{(PAH)}_i*{TEF}_i $$

**Table 2:** Mutagenic and carcinogenic equivalency factors for PAHs

PAH compound	Symbol	Mutagenic equivalency factor (MEF)^a^	Carcinogenic equivalency factor (TEF)^b^
Naphthalene	NP	–	0.001
Acenaphthene	ACE	–	0.001
Fluorene	FL	–	0.001
Phenanthrene	PHE	–	0.001
Anthracene	ANT	–	0.01
Fluoranthene	FLA	–	0.001
Pyrene	PYR	–	0.001
Benz(a)anthracene	BaA	0.082	0.1
Chrysene	CHR	0.017	0.01
Benzo(b)fluoranthene	BbF	0.25	0.1
Benzo(k)fluoranthene	BkF	0.11	0.1
Benzo(a)pyrene	BaP	1.0	1.0
Benzo(g,h,i)perylene	BghiP	0.19	0.01
Dibenz(a,h)anthracene	DahA	0.29	1.0
Indeno(1,2,3-c,d)pyrene	IcdP	0.31	0.1

^a^MEF according to Durant et al. (1996)

^b^TEF according to Nisbet and LaGoy (1992)

According to Delgado-Saborit et al. (2011) for 15 PAHs carcinogenic potential (CP) (in percent) was calculated (Eq. 3). Additionally, the contribution of the total mutagenicity of each of the eight PAH compounds expressed as mutagenic potential (MP) was determined using the following equation (Eq. 4):

3 $$ {CP}_i=\frac{\frac{(PAH)_i}{(BaP)}*{TEF}_i}{\sum_{i=1}^N\frac{(PAH)_i}{(BaP)}*{TEF}_i}*100\% $$

4 $$ {MP}_i=\frac{\frac{(PAH)_i}{(BaP)}*{MEF}_i}{\sum_{i=1}^N\frac{(PAH)_i}{(BaP)}*{MEF}_i}*100\% $$

### Statistical analysis

The obtained results were analyzed using the package Statistica for Windows, version 10. All analyzed variables did not have a normal distribution (Shapiro-Wilk test) even after logarithmic transformation. The median, minimum, and maximum values were used to describe the concentrations of PM2.5 and 15 PAHs and their sum. Due to the lack of normal distribution of the analyzed variables, caused by a limited number of samples, the differences between the two studied locations were calculated using the non-parametric Mann-Whitney test. *P* values ≤0.05 were considered to be statistically significant.

## Results

### PM2.5 and PAHs concentrations

The median with minimum and maximum values for PM2.5 and 15 PAHs and their sum concentrations observed in Dąbrowa Górnicza and Złoty Potok during spring season in 2010 are presented in Table 3. Twenty-four-hour concentrations of PM2.5 ranged from 22.8 to 45.0 μg/m^3^ and 16.0 to 69.5 μg/m^3^ in the outdoor air and from 18.5 to 42.4 μg/m^3^ and 20.0–41.9 μg/m^3^ in the indoor air of Dąbrowa Górnicza and Złoty Potok, respectively. The median for atmospheric PM2.5 was similar in two areas and exceeded the annual standard level for PM2.5 (25 μg/m^3^) (Directive 2008). Indoor concentration of PM2.5 was higher in Złoty Potok than in Dąbrowa Górnicza, but the difference was not statistically significant (Table 3).

**Table 3:** Indoor and outdoor 24-h concentrations of PM2.5, 15 PAHs, and their sum in two investigated sites in Silesia, Poland

Concentration (ng/m^3^)	Outdoor air					Indoor air				
	Dąbrowa Górniczas		Złoty Potok			Dąbrowa Górnicza		Złoty Potok		
	Median	Min-max	Median	Min-max	p*	Median	Min-max	Median	Min-max	p*
PM2.5^a^	32.5	22.8–45.0	30.6	16.0–69.5	0.438	25.1	18.5–42.4	36.1	20.0–41.9	0.535
NP	5.5	2.9–11.7	8.3^b^	5.3–9.8	0.211	4.7	2.9–7.0	4.9^b^	2.6–5.9	0.902
ACE	0.2	0.1–0.6	0.3	0.0–1.0	0.311	0.4	0.2–0.4	0.3	0.2–0.4	0.620
FL	1.1	0.6–2.3	1.2	0.2–1.7	0.485	0.8	0.6–1.1	1.0	0.4–2.0	0.165
PHE	4.6	1.6–13.7	2.9	0.1–11.6	0.485	3.5	1.1–9.2	4.2	2.3–8.0	0.805
ANT	1.1	0.3–2.7	0.5	0.2–2.2	0.311	0.7	0.2–1.6	0.6	0.3–2.0	0.902
FLA	5.1	1.4–10.1	3.0	1.1–11.7	0.817	3.9	1.2–7.8	3.2	1.6–10.0	0.805
PYR	5.3^b^	1.5–12.8	4.0^b^	1.6–11.7	0.536	2.6^b^	1.2–3.9	1.2^b^	1.0–2.6	0.053
BaA	4.7	1.0–15.6	2.9^b^	1.0–11.1	0.536	2.1	0.4–5.5	1.1^b^	0.5–4.2	1.000
CHR	4.7^b^	0.9–13.0	2.8	1.3–10.0	0.536	1.9^b^	0.6–4.9	1.5	0.7–4.6	1.000
BbF	4.9	1.8–8.1	3.1	1.7–8.9	0.211	3.7	1.4–6.6	3.1	2.1–9.9	0.805
BkF	2.5	0.8–4.4	1.6	0.7–6.0	0.351	1.8	0.6–3.4	1.6	1.1–5.4	0.805
BaP	4.0	1.1–8.0	3.1	1.0–8.1	0.351	3.6	1.2–7.5	3.1	2.7–12.8	0.710
BghiP	4.5	1.6–7.6	3.3	0.8–8.6	0.135	3.8	1.5–6.2	3.5	2.8–11.9	0.710
DahA	0.5	0.2–1.2	0.3	0.0–3.0	0.183	0.4	0.2–0.9	0.4	0.2–1.5	0.805
IcdP	4.4	1.8–7.4	2.6	1.1–8.5	0.351	3.7	1.4–7.4	3.9	2.7–11.2	0.535
Σ15 PAHs	52.9	18.0–117.7	39.3	22.8–107.8	0.588	36.1	14.6–72.9	31.4	25.0–89.5	1.000

^a^Concentration in μg/m^3^

^b^Statistically significant differences between outdoor vs. indoor concentrations

*Mann-Whitney test; differences between Dąbrowa Górnicza and Złoty Potok

For PM2.5-bound PAHs, statistical differences between Dąbrowa Górnicza and Złoty Potok were not significant either for outdoor or indoor samples. However, the highest 24-h concentration of 15 PAH sum (52.9 ng/m^3^) was detected in ambient air of Dąbrowa Górnicza (Table 3).

Generally, 24-h concentrations of outdoor PM2.5-bound BaP ranged from 1.1 to 8.0 ng/m^3^ for Dąbrowa Górnicza and from 1.0 to 8.1 ng/m^3^ for Złoty Potok, whereas the indoor PM2.5-bound BaP concentrations oscillated from 1.2 to 7.5 ng/m^3^ in Dąbrowa Górnicza and 2.7 to 12.8 ng/m^3^ in Złoty Potok. The median concentration of this compound was above 1 ng/m^3^ (Directive 2004). In Dąbrowa Górnicza, statistically higher values for PYR (*p* = 0.026) and CHR (*p* = 0.038) were observed in outdoor samples. In rural areas, statistically significant differences were found between outdoor and indoor air for NP (*p* ≤ 0.000), PYR (*p* ≤ 0.000), and BaA (*p* = 0.038) (Table 3).

### Mutagenicity and carcinogenicity of PAHs

The median values for mutagenic (MEQ) and carcinogenic (TEQ) equivalents related to the studied PAHs as well as, respectively - their mutagenic and carcinogenic potential expressed as a percentage are presented in Tables 5 and 6. Differences in MEQ and TEQ calculated for individual PAHs between Dąbrowa Górnicza and Złoty Potok were not statistically significant, both for outdoor and indoor samples. The summarized mutagenic equivalents (MEQ) for eight PAHs and carcinogenic equivalents (TEQ) for 15 PAHs were similar in all analyzed air samples. The obtained values for MEQ_Σ8 PAH_ ranged from 6.0 to 8.4 ng/m^3^ and for TEQ_Σ15 PAH_ - from 4.5 to 6.4 ng/m^3^ (Tables 5 and 6).

In the mutagenicity of outdoor and indoor air, the highest percentage share, like in other studies, belongs to BaP (Delgado-Saborit et al. 2011). This compound represented approximately 50% of the whole mutagenic potential (MP) for eight investigated PAHs in samples collected in two locations. Several percent shares in the mutagenic potential was observed for IcdP, BbF, and BghiP, whereas the activity of the remaining compounds was inconsiderable (Table 5).

In the studied areas, the highest percentage share in carcinogenic potential (CP) was recorded in the case of BaP (above 60%), both in outdoor and indoor samples. The share of such compounds as BaA, BbF, DahA, and IcdP in carcinogenic activity of the studied air samples was at the level of a few percent and the share of other compounds was insignificant (Table 6).

## Discussion

### PM2.5 concentrations

Analysis of outdoor PM2.5 concentrations in the Silesian region that have been carried out since 2001 indicate the lack of strong downward trend in the average annual concentration and the dominant influence of the winter season. High annual concentrations of PM2.5 in the Silesian cities can be attributed to high concentrations of PM2.5 in winter (3 to 5 times higher than in summer) (Rogula-Kozłowska et al. 2014). It has been shown earlier that the main reason for the occurrence of the exceeded levels for outdoor PM2.5 and BaP in the winter season in southern Poland was mainly the emission connected with individual heating of buildings and to a lower degree - road transport, metrological conditions, as well as transboundary movement of air pollutants (Rogula-Kozłowska et al. 2014). In the carried out study, the ambient concentrations of PM2.5 were above 30.0 μg/m^3^ in two investigated locations (Table 3). The higher levels of this pollutant (exceeding 40.0 μg/m^3^) were observed during winter season in Zabrze, Katowice (2005–2007; 2009–2010), and Racibórz (2011–2012) (Klejnowski et al. 2009; Rogula-Kozłowska et al. 2014, 2016).

Concentration at a similar level as in the present study was obtained in atmospheric air during winter in Praha (27.3 μg/m^3^) where emission from coal combustion dominates (Gotschi et al. 2002). However, the highest outdoor levels of PM2.5 were observed in Hong Kong (range 42.0–197.2 μg/m^3^) and a Chinese city of Guangzhou (123.7 μg/m^3^) representing an urban area with high traffic flow (Ho et al. 2004; Huang et al. 2007).

In this study, indoor concentrations of PM2.5 were 25.1 μg/m^3^ in the urban area (Dąbrowa Górnicza) and 36.1 μg/m^3^ in the rural site (Złoty Potok) (Table 3). Higher level of indoor PM2.5 in the rural area in comparison to the urban site, but not statistically significant, can be observed due to the use of coal stove in a kindergarten kitchen (Table 1). In kindergartens of southern Poland during winter season 2013/2014, the indoor level of PM2.5 was higher in rural (94.1 μg/m^3^) than in urban (66.7 μg/m^3^) locations. What is more, the worst indoor air quality was observed in a nursery school situated next to the highway and in older children’s classrooms in the two studied areas (Mainka and Zajusz-Zubek 2015). In many studies, concentration of PM2.5 is associated with outdoor penetration as well as compensation of indoor sources related to the presence of children and the intensity of their indoor activity even if the detected values are low, as it was measured in four urban elementary schools in Cincinnati (15.6 μg/m^3^), ten kindergartens (6.1 μg/m^3^), six schools in Stockholm (8.1 μg/m^3^), and also primary schools in Brisbane (6.7 μg/m^3^) (Guo et al. 2010; Wichmann et al. 2010; Hochstetler et al. 2011). A much higher level of PM2.5 was observed in indoor air samples collected during winter time in schools of Munich (38.9 μg/m^3^) which was corresponding to a small room size and a high number of occupants (Fromme et al. 2007).

The obtained results show that differences between indoor and outdoor levels of PM2.5 were not statistically significant either in Dąbrowa Górnicza or Złoty Potok. Comparable data were also found in studies carried out in 24 schools near motorways in the Netherlands (Janssen et al. 2001). Outdoor PM2.5 concentrations were similar to indoor levels in primary school classrooms in Lisbon, but the analysis of PM2.5–10 fraction showed statistical differences and indoor higher levels of this fraction. Therefore, the authors suggest that occupancy, through re-suspension of previously deposited particles and possibly their generation, strongly influences the indoor concentration level of airborne particles (Almeida et al. 2011). Thus, it can be concluded that outdoor air quality has an impact on indoor air, especially in regions with high PM2.5 ambient concentrations, but human activity, as well as internal sources may have a dominant influence on indoor microenvironments in particular with natural ventilation systems. Typical rural areas located next to industrial areas or highways seem to be more at risk due to usually older building construction with traditional cooking and heating coal stoves.

### PAHs concentrations

Although outdoor 24-h concentrations of PM2.5-bound BaP collected in two investigated locations were above 3.0 ng/m^3^ (Table 3), the air quality in the Silesian region has improved in comparison to the previous years. A lower level of PM-bound BaP was observed during winter–spring time in southern Taiwan (1.7 ng/m^3^) (Lin et al. 2002). Ambient concentrations of PM2.5-bound BaP equal or below 0.1 ng/m^3^ were found in Los Angeles, Houston, and North Carolina (Naumova et al. 2002; Wilson et al. 2003). Winter and summer outdoor concentrations of PM2.5-bound PAH in New York were 0.12 and 0.05 ng/m^3^, respectively (Jung et al. 2010). The data presented above suggest that BaP outdoor concentrations measured in Silesian areas are higher than in North America or other European countries. This situation is connected with coal or wood combustion in private heating systems and industrial energy sectors, which can be reflected in concentrations and chemical composition of PM (Rogula-Kozłowska 2016).

Concentration of PM2.5-bound BaP inside kindergartens or schools is rarely examined. However, as shown in this study, indoor concentrations of BaP in two kindergartens in southern Poland were high (Table 3) and exceeded 1 ng/m^3^ (Directive 2004). In European cities, lower indoor air levels of BaP were recorded in schools during winter season in the city of Rome (1.13 ng/m^3^) (Romagnoli et al. 2014). In studies of children’s exposure in New York, the level of this compound was 0.14 ng/m^3^ and 0.06 ng/m^3^ during cold (heating) and warm (non-heating) season, respectively (Jung et al. 2010).

In this study, the sum of 15 investigated PAHs in outdoor PM2.5 air samples ranged from 18.0 to 117.7 ng/m^3^, with median 52.9 ng/m^3^ in Dąbrowa Górnicza and 39.3 ng/m^3^ in Złoty Potok (Table 3). Also, in Złoty Potok during winter 2009/2010 (heating period), the sum of 16 PM2.5-bound PAHs was 56.8 ng/m^3^, while at the same time in Katowice, it was six times higher (Rogula-Kozłowska et al. 2013a). The winter (2006/2007) ambient concentrations of 15 PAHs in Zabrze, also in PM2.5 fraction, were high - 153.9 ng/m^3^ (Rogula-Kozłowska et al. 2013b). However, extremely high levels for Σ13 PAHs in atmospheric air samples were measured in Częstochowa (367.9 ng/m^3^) and the values exceeding 600 ng/m^3^ were recorded in Bytom, Sosnowiec, and Katowice (Kozłowska et al. 2011). All this data suggest that in Poland, the outdoor PM2.5 air samples collected in big cities contain more PAHs than in rural sites, which can be connected with the increased number of industrial emission sources. Therefore, southern Poland represents one of the most polluted areas in Europe, which should be considered as a “hot spot” and highly monitored.

The sum of the 15 studied PAHs in indoor PM2.5 samples collected in two kindergartens was above 30.0 ng/m^3^ (Table 3). Similar concentrations were observed in Lithuanian primary schools (Σ15 PAHs range 20.3–131.1 ng/m^3^) (Krugly et al. 2014). Concentrations of the sum of 12 selected PAHs in kindergartens in Montreal were lower both in polluted (3.0 ng/m^3^) and control areas (2.1 ng/m^3^) (Gatto et al. 2014). Also, in two Portugal kindergartens, indoor concentrations of the sum of 16 PAHs were 1.4 and 4.2 ng/m^3^ (Oliveira et al. 2016). Trends in PAH concentrations are characterized by site or country specificity, which is mainly connected with a typical factor such as emission source and additional factors such as long distance transport of pollutants, ventilation systems in buildings and human activity, etc. In this study, the lack of significant differences in the concentrations of the 15 investigated PM2.5-bound PAHs, both in outdoor and indoor air, may suggest that they are most likely affected by the same major sources.

### PAH diagnostic ratios

To identify the possible origin of PAHs, the diagnostic ratios calculated on the basis of the individual PM2.5-bound PAH concentrations were used. Results for outdoor and indoor PM2.5 sampling in Dąbrowa Górnicza and Złoty Potok are presented in Table 4. At both locations, the ratio of two- and three-ring PAHs (the total low molecular weight PAHs; ΣLMW) to four- and five-ring PAHs (the total high molecular weight PAHs; ΣHMW) was lower than 1, which might suggest pyrogenic source of PAHs. The same kind of PAH source was indicated by the value of ANT/(ANT + PHE), both for outdoor and indoor samples (Rogula-Kozłowska 2015). It should be mentioned that the low molecular weight PAHs dominantly occurred in the gaseous phase, which was not examined in this study. But in Poland during spring season, when the average temperature does not exceed 10 °C, PAH compounds usually bound to particulate matter (Rogula-Kozłowska 2016). The FLA/(FLA + PYR) ratio outside the kindergartens suggests fossil fuel combustion (range 0.4–0.5) as the PAH source. Higher values of FLA/(FLA + PYR) ratio (>0.5) inside the kindergartens could be an effect of compensation of outdoor and indoor pollutants from wood or coal combustion as well as a natural ventilation system in both studied buildings. Values close to 0.5 for BaA/(BaA + CHR) and IcdP/(IcdP + BghiP) inside and outside the kindergartens demonstrate that PM2.5-bound PAHs derive mostly from coal combustion (Rogula-Kozłowska 2015).

**Table 4:** Diagnostic ratios for PM2.5-bound PAHs in indoor and outdoor air collected in two investigated sites in Silesia, Poland

Ratio^a^	Outdoor air		Indoor air		Range	Source type
	Dąbrowa Górnicza	Złoty Potok	Dąbrowa Górnicza	Złoty Potok		
ΣLMW/ΣHMW	0.47	0.68	0.62	0.62	<1	Pyrogenic
FL/(FL + PYR)	0.19	0.22	0.27	0.41	<0.5	Petrol emission
ANT/(ANT + PHE)	0.17	0.20	0.15	0.14	>0.1	Pyrogenic
PHE/PHE + ANT	0.83	0.80	0.85	0.86	0.76	Coal
FLA/(FLA + PYR)	0.45	0.46	0.60	0.70	0.4–0.5	Fossil fuel combustion
					>0.5	Grass, wood, and coal combustion
					0.57	Coal burning
BaA/(BaA + CHR)	0.51	0.50	0.49	0.47	0.5	Coal/coke
					0.46	Coal burning
BaP/(BaP + CHR)	0.47	0.48	0.67	0.73	0.5	Diesel
					0.73	Gasoline
BaA/BaP	1.19	1.13	0.49	0.34	0.5	Gasoline
					1	Diesel and wood combustion
IcdP/(IcdP + BghiP)	0.49	0.50	0.50	0.51	0.2–0.5	Petroleum combustion
					>0.5	Grass, wood and coal combustion
IcdP/BghiP	0.98	1.08	1.00	1.03	~1	Diesel
BbF/BkF	1.97	1.96	2.01	1.91	>0.5	Diesel
BaP/BghiP	0.91	1.06	0.98	1.00	0.9–6.6	Wood combustion

*ΣLMW* total concentrations of two- and three-ring PAHs, *ΣHMW* total concentrations of four- and five-ring PAHs

^a^PAH diagnostic ratios for various sources adopted from Tables 3 and 4 in Rogula-Kozłowska (2015) and references cited therein

However, apart from fossil fuels (or/and biomass), the emission from road traffic was also identified. It was confirmed by FL/(FL + PYR) ratios lower than 0.5 (petroleum emission), IcdP/BghiP close to 1, and BbF/BkF higher than 0.5 (diesel combustion) (Rogula-Kozłowska 2015). Similar origin of indoor and outdoor PM2.5-bound PAHs in the two studied areas may suggest that most of PM2.5-bound PAHs might have penetrated from the atmospheric air into the kindergartens. This, however, should be clearly confirmed by measurements of air exchange ratios, which unfortunately were not included in this study (Table 4).

Our results indicate that both fossil fuels (mainly hard or brown coal) and road traffic are the main sources of PM2.5-bound PAH both at urban and rural areas and they are consistent with the previous studies (Rogula-Kozłowska et al. 2013a).

### Mutagenicity and carcinogenicity of PAHs

In this study, comparable levels for outdoor MEQ_Σ8 PAH_ were detected in urban (8.4 ng/m^3^) and rural (6.0 ng/m^3^) areas in the Silesian region (Table 5). However, in other Polish studies carried out in 2009/2010, authors demonstrated differences for MEQ between rural (8.1 ng/m^3^) and urban areas (24.4 ng/m^3^) (Rogula-Kozłowska et al. 2013a). Similar results for PM2.5 air samples were obtained in European countries, such as Lithuania (4.1–19.2 ng/m^3^) and northern Italy (4.2 ng/m^3^) (Masiol et al. 2012; Krugly et al. 2014). However, higher MEQ (27.0 ng/m^3^) showed air samples of PM2.5 collected in Teheran, which may be related to a heavy vehicular traffic flow and emissions from the nearby industries (Hassanvand et al. 2015).

**Table 5:** Mutagenic equivalent (MEQ) and mutagenic potential (MP) of outdoor and indoor samples collected in two investigated sites in Silesia, Poland

PAHs compounds	Outdoor air					Indoor air				
	Dąbrowa Górnicza		Złoty Potok			Dąbrowa Górnicza		Złoty Potok		
	MEQ (ng/m^3^)	MP (%)	MEQ (ng/m^3^)	MP (%)	*p* ^b^	MEQ (ng/m^3^)	MP (%)	MEQ (ng/m^3^)	MP (%)	*p* ^b^
BaA	0.4	4.0	0.2	4.0	0.536	0.2	2.0	0.1	1.5	1.000
CHR	0.1	1.0	0.0	1.0	0.536	0.0	0.0	0.0	0.0	1.000
BbF	1.2	14.0	0.8	14.0	0.211	0.9	13.0	0.8	11.0	0.805
BkF	0.3	3.0	0.2	3.0	0.351	0.2	3.0	0.2	3.0	0.805
BaP	4.0	49.0	3.1	50.5	0.351	3.6	53.0	3.1	54.5	0.710
BghiP	0.9	10.0	0.6	10.0	0.135	0.7	10.0	0.7	10.5	0.710
DahA	0.2	2.0	0.1	2.0	0.183	0.1	2.0	0.1	2.0	0.805
IcdP	1.4	17.0	0.8	15.5	0.351	1.1	17.0	1.2	17.5	0.535
Σ8 PAHs^a^	8.4	100.0	6.0	100.0	0.351	6.7	100.0	6.1	100.0	0.710

^a^Benz(a)anthracene, chrysene, benzo(b)fluoranthene, benzo(k)fluoranthene, benzo(a)pyrene, benzo(g,h,i)perylene, dibenz(a,h)anthracene, indeno(1,2,3-c,d)pyrene

^b^Mann-Whitney test; differences of MEQ between Dąbrowa Górnicza and Złoty Potok

Indoor MEQ calculated for eight PAHs in the two investigated sites was around 6.0 ng/m^3^ (Table 6). Similar levels for this parameter in indoor air were found in Lithuanian schools (0.8–14.6 ng/m^3^) (Krugly et al. 2014). However, PM2.5 collected in a boarding school in Teheran was higher (22.0 ng/m^3^), but this level may be associated with high outdoor PAH concentrations (Hassanvand et al. 2015).

**Table 6:** Carcinogenic equivalent (TEQ) and carcinogenic potential (CP) of outdoor and indoor samples collected at two sites in Silesia, Poland

PAHs compounds	Outdoor air					Indoor air				
	Dąbrowa Górnicza		Złoty Potok			Dąbrowa Górnicza		Złoty Potok		
	TEQ (ng/m^3^)	CP (%)	TEQ (ng/m^3^)	CP (%)	*p* ^b^	TEQ (ng/m^3^)	CP (%)	TEQ (ng/m^3^)	CP (%)	*p* ^b^
NP	0.0	0.0	0.0	0.0	0.211	0.0	0.0	0.0	0.0	0.902
ACE	0.0	0.0	0.0	0.0	0.311	0.0	0.0	0.0	0.0	0.620
FL	0.0	0.0	0.0	0.0	0.485	0.0	0.0	0.0	0.0	0.165
PHE	0.0	0.5	0.0	0.0	0.485	0.0	0.0	0.0	0.5	0.805
ANT	0.0	0.0	0.0	0.0	0.311	0.0	0.0	0.0	0.0	0.902
FLA	0.0	0.0	0.0	0.0	0.817	0.0	0.0	0.0	0.0	0.805
PYR	0.0	0.0	0.0	0.0	0.536	0.0	0.0	0.0	0.0	0.053
BaA	0.5	7.0	0.3	7.0	0.536	0.2	3.5	0.1	3.0	1.000
CHR	0.0	1.0	0.0	1.0	0.536	0.0	0.5	0.0	0.5	1.000
BbF	0.5	8.0	0.3	7.0	0.211	0.4	7.0	0.3	6.0	0.805
BkF	0.2	4.0	0.2	4.0	0.351	0.2	3.5	0.2	3.0	0.805
BaP	4.0	62.5	3.1	66.0	0.351	3.6	69.0	3.1	70.0	0.710
BghiP	0.0	1.0	0.0	1.0	0.135	0.0	1.0	0.0	1.0	0.710
DahA	0.5	9.0	0.3	7.0	0.183	0.4	8.5	0.4	8.0	0.805
IcdP	0.4	7.0	0.3	7.0	0.351	0.4	7.0	0.4	8.0	0.535
Σ15 PAHs^a^	6.4	100.0	4.6	100.0	0.351	5.2	100.0	4.5	100.0	0.710

^a^Naphthalene, acenaphthene, fluorine, phenanthrene, anthracene, fluoranthene, pyrene, benz(a)anthracene, chrysene, benzo(b)fluoranthene, benzo(k)fluoranthene, benzo(a)pyrene, benzo(g,h,i)perylene, dibenz(a,h)anthracene, and indeno(1,2,3-c,d)pyrene

^b^Mann-Whitney test; differences of TEQ between Dąbrowa Górnicza and Złoty Potok

Carcinogenic equivalent (TEQ) calculated based on the concentration of 15 PAHs bound to PM2.5 in outdoor air was 6.4 ng/m^3^ in Dąbrowa Górnicza and 4.6 ng/m^3^ in Złoty Potok (Table 6). Similar data were observed for ambient PM2.5 during winter in north Italy (TEQ_Σ11 PAH_ 3.6 ng/m^3^) (Masiol et al. 2012). In the atmospheric air (PM2.5) of Shizuoka (Japan), TEQ was lower, 0.7 ng/m^3^ (Ohura et al. 2005). Higher levels of TEQ were detected for PM2.5 in Lithuania and in the capital of Iran (Krugly et al. 2014; Hassanvand et al. 2015).

In this study, the TEQ_Σ15 PAH_ assessed for indoor air samples was similar to the values of this equivalent calculated for ambient air in the two studied areas (Table 6). In individual samples of PM2.5 taken indoors during winter season in Japan, the level of TEQ was lower (~0.5 ng/m^3^), both in cold and warm period (Ohura et al. 2005). Higher TEQ values calculated for the same air fraction were found in Lithuanian schools (1.2–50.8 ng/m^3^) and a boarding school in Teheran (26.2 ng/m^3^) (Krugly et al. 2014; Hassanvand et al. 2015).

In this study, the highest percentage share in MEQ and TEQ calculated for indoor and outdoor PM2.5 samples belongs to BaP, which was previously documented (Ohura et al. 2005). These results suggest the suitability of BaP as a marker for the mutagenic and carcinogenic potential of the environmental PAH mixtures. High levels for all the obtained equivalents indicate insufficient ventilation in both kindergartens resulting in poor indoor air quality and may cause PAH-related adverse health effects. It should emphasized here that MEQ and TEQ values obtained in this study are underestimated because not all the PAHs have been analyzed. In addition, some of the PAH derivatives (e.g., nitro-PAH) are more harmful than the parent compounds.

## Conclusions

In developing countries as well as in industrialized regions, such as, for example, southern Poland, the air quality has a large impact on the health of individuals and the society as a whole and is still a big challenge.

Results of this study indicate that PM2.5 and PM2.5-bound PAHs occurring both in the air outside and inside the kindergartens in the Silesian region are an important source of children’s exposure to genotoxic agents. High levels of PM2.5-bound PAHs are characteristic for winter (heating period) as a result of local emission - coal and biomass combustion in home furnaces and increased production in heating or/and power plants. Concentrations of PM-bound PAHs in Złoty Potok and Dąbrowa Górnicza are comparable to concentrations recorded in big agglomerations/cities in Asia. The impact of airborne particulate matter on human health has been extensively studied. It is well known that this pollutant can induce airway inflammation, implicate oxidative stress, and impair antioxidant defenses as risk factors. These facts suggest that air particles in ambient air even in low concentrations may activate non-genotoxic (epigenetic) mechanisms. In such situations, the action of BaP and other PAHs adsorbed onto particulates could well be a more significant risk factor.

Approaches such as mutagenic (MEQ) and carcinogenic (TEQ) equivalents in the recent years have become more often used in exposure studies. These tools take into consideration not only the concentration of the investigated compounds but also their biological activity, which enables more adequate assessment. What is more, mutagenic (MP) and carcinogenic (CP) potential describes the quantitative contribution related to BaP, which can be helpful in drawing conclusions.
